# Ocular Decompression Retinopathy Followed by Central Retinal Vein Occlusion After Augmented Trabeculectomy in Pseudoexfoliative Glaucoma: A Case Report

**DOI:** 10.7759/cureus.112968

**Published:** 2026-07-19

**Authors:** Shahrir Fadhli Fakhrurazi, Teck Chee Cheng, Safinaz Mohd Khialdin, Jemaima Che Hamzah

**Affiliations:** 1 Department of Ophthalmology, Faculty of Medicine, Universiti Kebangsaan Malaysia, Kuala Lumpur, MYS

**Keywords:** augmented trabeculectomy, central retinal vein occlusion, cystoid macular edema, ocular decompression retinopathy, pseudoexfoliative glaucoma

## Abstract

We report a rare sequential presentation of ocular decompressive retinopathy (ODR) and central retinal vein occlusion (CRVO) following trabeculectomy in a patient with pseudoexfoliative glaucoma (PXG). A 62-year-old woman with poorly controlled hypertension, bilateral grade II hypertensive retinopathy, and left-eye PXG initially achieved intraocular pressure (IOP) control with maximal medical therapy but was lost to follow-up. She re-presented with an IOP of 65 mmHg in the left eye and progressive glaucomatous damage despite maximal topical and systemic agents, necessitating augmented trabeculectomy with mitomycin C. Postoperatively, she developed hypotony (IOP: 5 mmHg) with fundus findings of ocular decompression retinopathy. Six weeks later, vision declined from 6/18 to 2/60 with new dilated tortuous veins, worsening hemorrhages, and cystoid macular edema (CME) confirmed on optical coherence tomography (OCT) (central subfield thickness (CST): 1100 µm). Fundus fluorescein angiography (FFA) showed delayed arteriovenous (AV) transit without capillary nonperfusion, consistent with nonischemic CRVO. She received three monthly intravitreal ranibizumab injections, resulting in the best-corrected visual acuity (BCVA) improvement of 6/24, marked CME resolution (389 µm), and stable IOP in the low teens. This case highlights that CRVO may occur after ODR-like postoperative retinal hemorrhages in high-risk patients after trabeculectomy, emphasizing the need for vigilant postoperative surveillance and prompt intervention to preserve vision.

## Introduction

First reported by Fechtner et al., ocular decompression retinopathy (ODR) is a rare condition triggered by rapid iatrogenic lowering of intraocular pressure (IOP), often occurring as a complication of glaucoma filtering surgery [1]. The abrupt reduction in IOP leads to acute ocular decompression, manifesting as a spectrum of retinal hemorrhages and optic nerve changes.

Clinically, about 80% of patients are asymptomatic. Symptomatic patients typically present with decreased vision, central scotoma, or floaters [2]. Fundoscopic examination typically reveals scattered superficial and deep intraretinal blot hemorrhages, often with white-centered lesions, predominantly in the posterior pole and equatorial retina. Optic disc changes include peripapillary hemorrhages, hyperemia, and disc edema. Subhyaloid or vitreous hemorrhages occur less frequently but are occasionally observed. Retinal vessels generally appear normal, with no significant venous tortuosity or dilatation. Choroidal detachment and macula edema are rare.

It is crucial to clinically distinguish ODR from central retinal vein occlusion (CRVO). The diffuse, multilayered retinal hemorrhages observed in ODR can resemble those seen in CRVO. However, ODR typically presents in younger patients, is often asymptomatic, and is associated with full recovery of visual acuity. Unlike CRVO, ODR rarely exhibits venous dilatation or delayed venous filling on fluorescein angiography. Additionally, persistent macular edema, a hallmark of CRVO, has not been reported in ODR cases, which require prompt antivascular endothelial growth factor (anti-VEGF) therapy.

This case report details a patient with pseudoexfoliative glaucoma (PXG) and uncontrolled IOP who underwent augmented trabeculectomy, which later developed ODR followed by subsequent CRVO. We emphasize the diagnostic challenges, management strategies, and clinical implications of this uncommon complication, underscoring the importance of vigilant postoperative monitoring in high-risk patients.

## Case presentation

**Figure 1:**
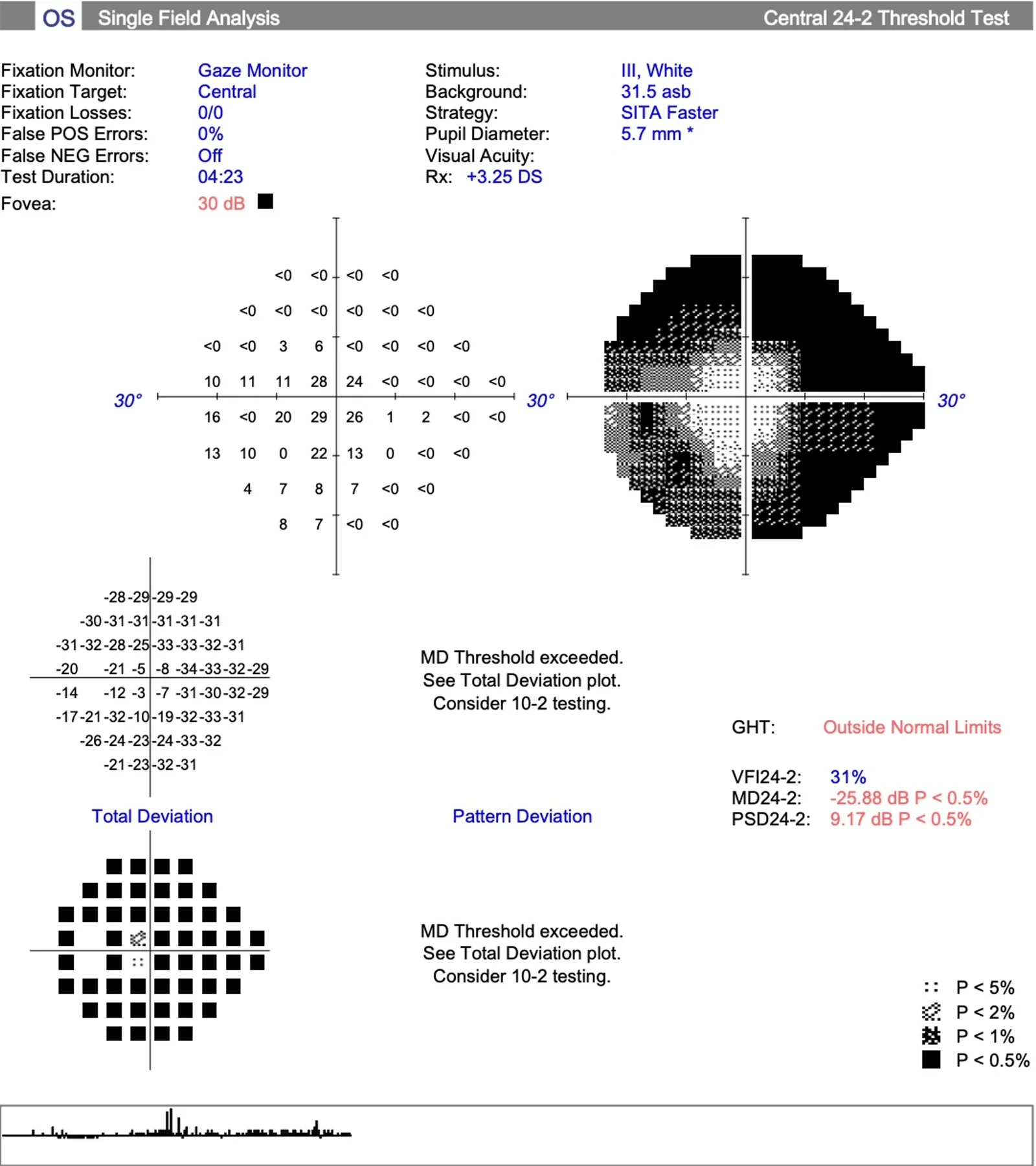
Humphrey visual field 24-2 of the left eye showing tunnel vision

A 62-year-old woman with poorly controlled hypertension had been under ophthalmic follow-up for two years for left eye PXG, bilateral grade II hypertensive retinopathy, and bilateral immature cataracts. Throughout the follow-up period, her blood pressure remained suboptimally controlled, ranging from 143-176 mmHg systolic and 79-93 mmHg diastolic, with inconsistent medical follow-up. She also had concomitant dyslipidemia, with elevated triglycerides (3.7 mmol/L) and low-density lipoprotein cholesterol (4.9 mmol/L). She was a nonsmoker, had no history of cardiovascular disease, and was not diabetic. Her presenting IOP was 62 mmHg in the left eye with clinical features suggestive of pseudoexfoliation syndrome, including pseudoexfoliative material at the pupillary margin, a hoarfrost ring on the anterior lens capsule, and a Sampaolesi line on gonioscopy. The best-corrected visual acuity (BCVA) was 6/6 in both eyes with a positive relative afferent pupillary defect (RAPD) on the left. Fundus examination revealed a glaucomatous optic disc with a cup-to-disc ratio (CDR) of 0.8 in the left eye, while the right eye had a normal IOP and CDR of 0.3. Retinal vessels in both eyes exhibited arteriovenous (AV) nicking and copper wiring, consistent with hypertensive retinopathy. Visual field testing in the left eye demonstrated advanced glaucoma with tunnel vision (Figure 1).

She was initially treated with regular oral acetazolamide and subsequently maintained an IOP at 15-20 mmHg on five topical antiglaucoma eyedrops including latanoprost, brinzolamide, timolol, brimonidine, and ripasudil. Although advised to undergo filtering surgery, she declined and was subsequently lost to follow-up. After one year without review and four months off all topical antiglaucoma, she re-presented with an IOP of 65 mmHg in the left eye. Her BCVA was unchanged, but the optic disc revealed further cupping with confirmed progression on visual field tests. Maximal medical therapy comprising five topical agents, oral acetazolamide 250mg four times daily, and syrup glycerol 100% 20 mL three times daily reduced IOP to only 26 mmHg.

She subsequently underwent trabeculectomy augmented with mitomycin C (MMC) 0.02% under local anesthesia. A fornix-based conjunctival peritomy was created, followed by dissection of a partial-thickness scleral flap. A cellulose sponge soaked with MMC (0.2 mg/mL) was applied beneath the scleral flap for three minutes before thorough irrigation. A slow paracentesis was performed, and an anterior chamber maintainer was inserted. Sclerostomy and peripheral iridectomy were then completed. The scleral flap was secured with four interrupted 10-0 nylon sutures, and the Tenon's capsule and conjunctiva were closed with 10-0 nylon sutures. The procedure was uneventful, and all topical antiglaucoma medications were discontinued postoperatively.

**Figure 2:**
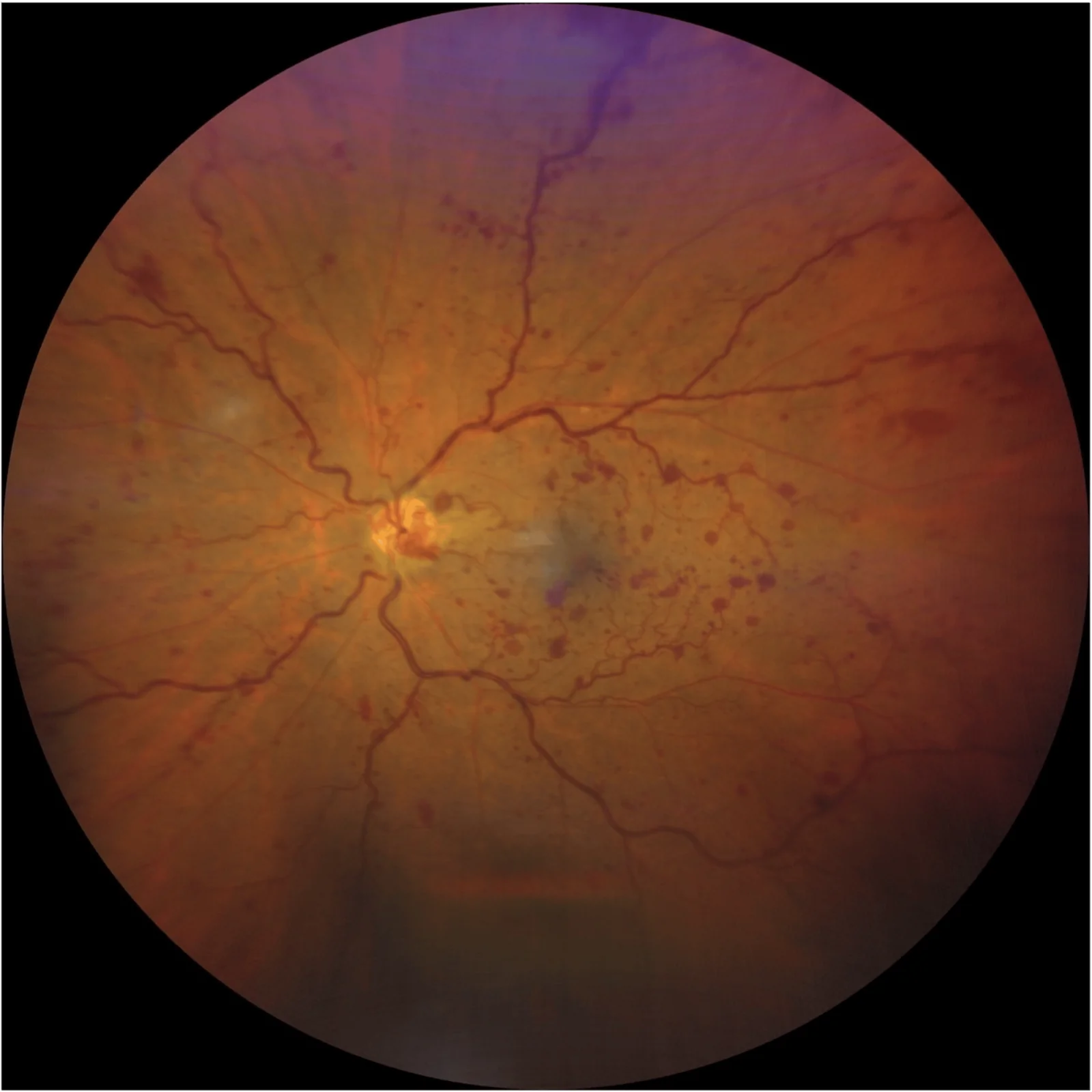
Fundus photo of the left eye showing hyperemic swollen optic disc, scattered superficial and deep blot hemorrhages with some white-centered hemorrhages located at posterior pole and mid-peripheral retina. The retinal veins were mildly tortuous but not dilated

On postoperative day one, the patient developed numerical hypotony (IOP: 5 mmHg) secondary to overfiltration. Slit-lamp examination demonstrated an elevated filtering bleb with a slow leak identified at the conjunctival suture site. Her anterior chamber remained deep with no sequelae of hypotony such as choroidal detachment or hypotony maculopathy. Her fundus examination revealed a hyperemic, swollen optic disc, scattered superficial and deep blot hemorrhages with some white-centered hemorrhages located at the posterior pole and mid-peripheral retina. The retinal veins were mildly tortuous but not dilated. Optical coherence tomography (OCT) of her left macula showed a normal foveal contour. These features were consistent with ODR (Figure 2) rather than early CRVO due to multilayered hemorrhages after rapid IOP reduction, swollen disc, absence of marked venous dilatation, normal foveal contour on OCT, and no initial macular edema. She was managed with a tapering regimen of topical corticosteroids, and a bandage contact lens was applied to promote conjunctival wound healing, resulting in complete resolution of the leak by postoperative day four.

**Figure 3:**
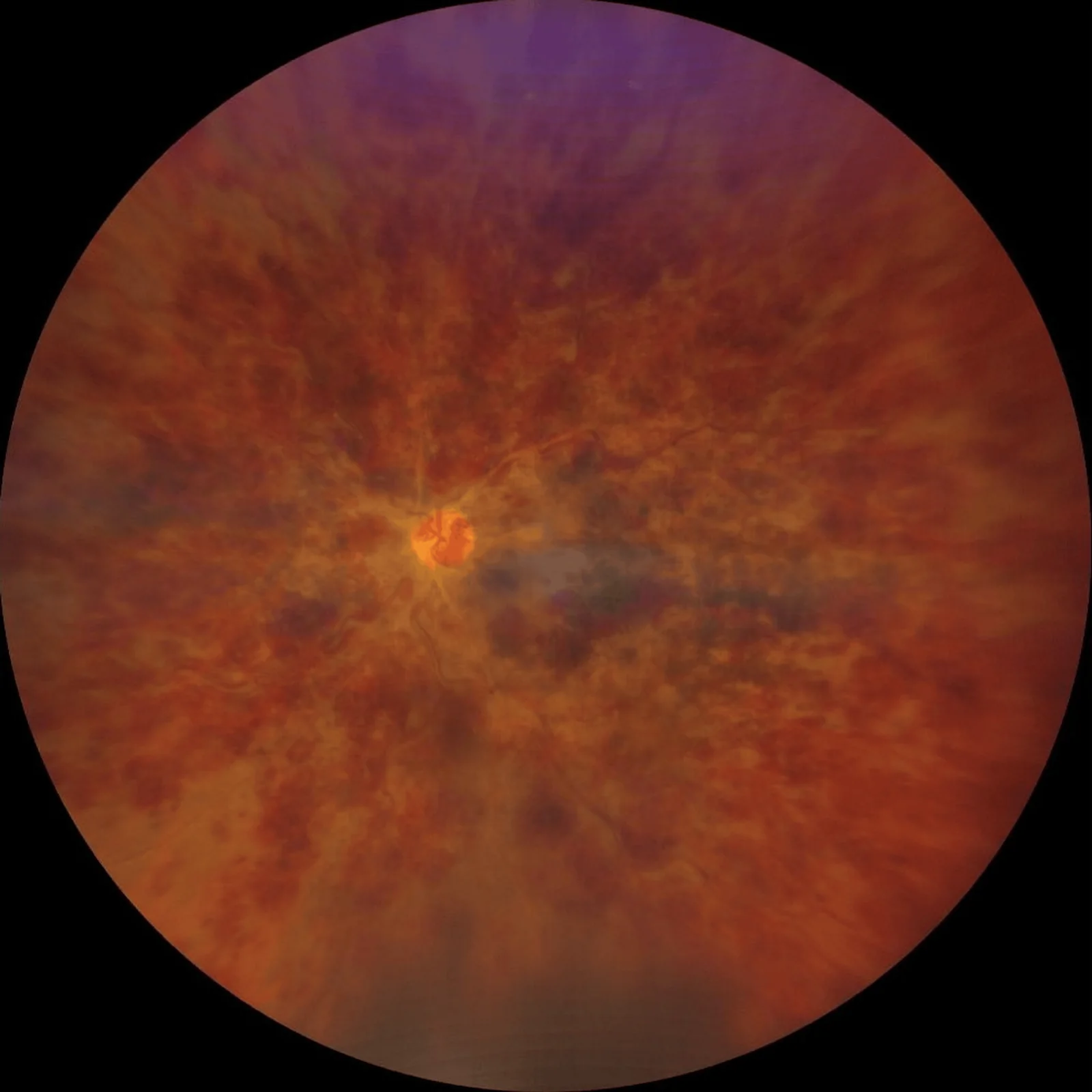
Fundus photo of the left eye showing hyperemic disc with optociliary shunt, worsening blot and flame-shaped hemorrhages in all quadrants, tortuous and dilated veins and a dull foveal reflex, suggestive of central retinal vein occlusion with cystoid macula edema

**Figure 4:**
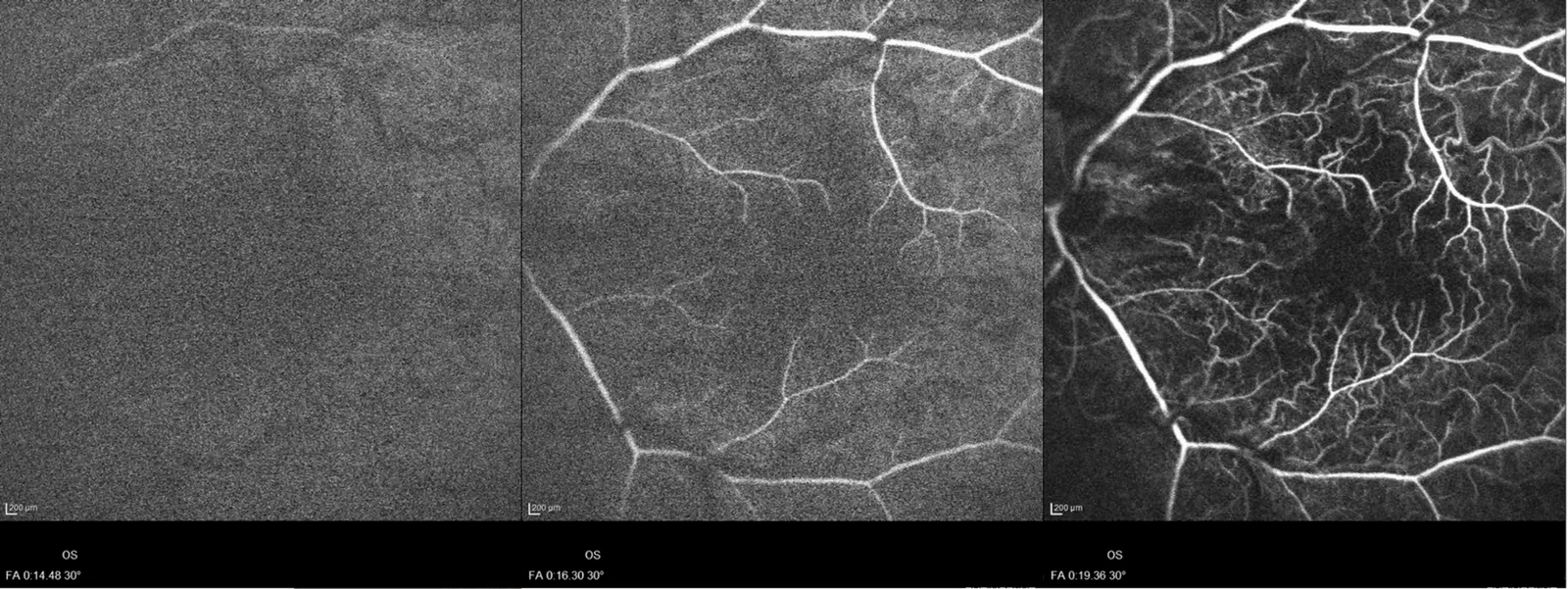
Fundus fluorescein angiography of the left eye showing delayed arteriovenous transit. Top row (left to right): early choroidal phase (~14 s), arteriovenous phase (~16 s) and early venous phase with initial venous filling (~19 s). Extensive retinal hemorrhages masked portions of the peripheral retina, limiting complete assessment of subtle nonperfusion

At six weeks postoperatively, her BCVA declined from 6/18 to 2/60. Her bleb remained well-elevated with deep anterior chamber and stable IOP at 9 mmHg. Funduscopy revealed a hyperemic disc with optociliary shunt, worsening blot and flame-shaped hemorrhages in all quadrants, tortuous and dilated veins, and a dull foveal reflex, suggestive of CRVO with cystoid macula edema (CME) (Figure 3). She did not have rubeosis iridis or any neovascularization. OCT confirmed CME with a central subfield thickness (CST) of 1100 μm. Fundus fluorescein angiography (FFA) demonstrated delayed AV transit time, with initial venous filling observed at 19 seconds and a classic petaloid leakage pattern in the macular region, consistent with CME. Hypofluorescence areas corresponded to the masking effect of intraretinal hemorrhages. Notably, there was no evidence of capillary nonperfusion or ischemia (Figure 4). These findings favored nonischemic CRVO, given reduced BCVA, dilated, tortuous veins, hemorrhages in all quadrants, marked CME, and delayed AV transit time on FFA.

**Figure 5:**
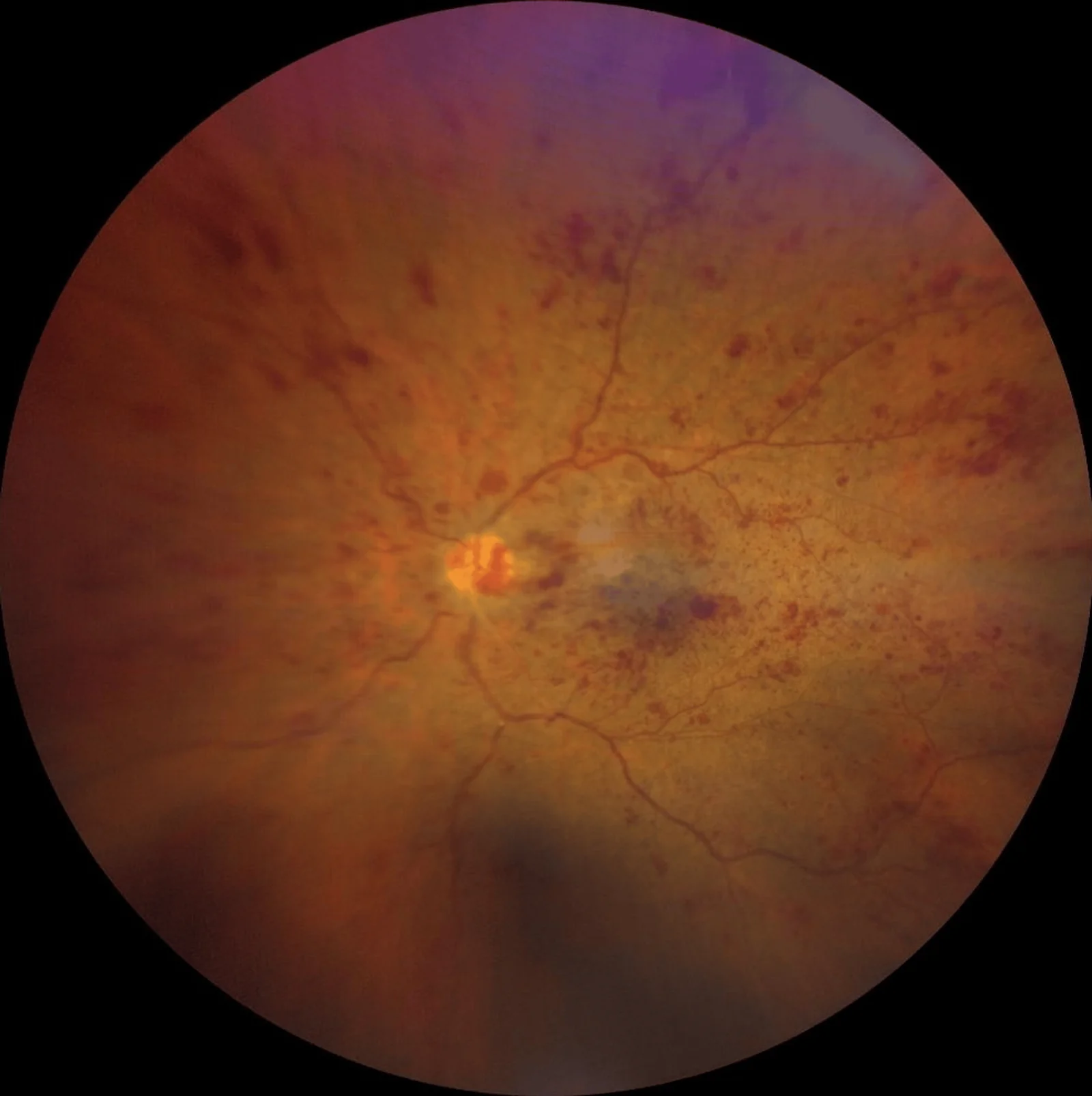
Fundus photo of the left eye showing pink optic disc with resolving blot and flame shape hemorrhages and no signs of ischemic conversion such as neovascularization and vitreous hemorrhage

**Figure 6:**
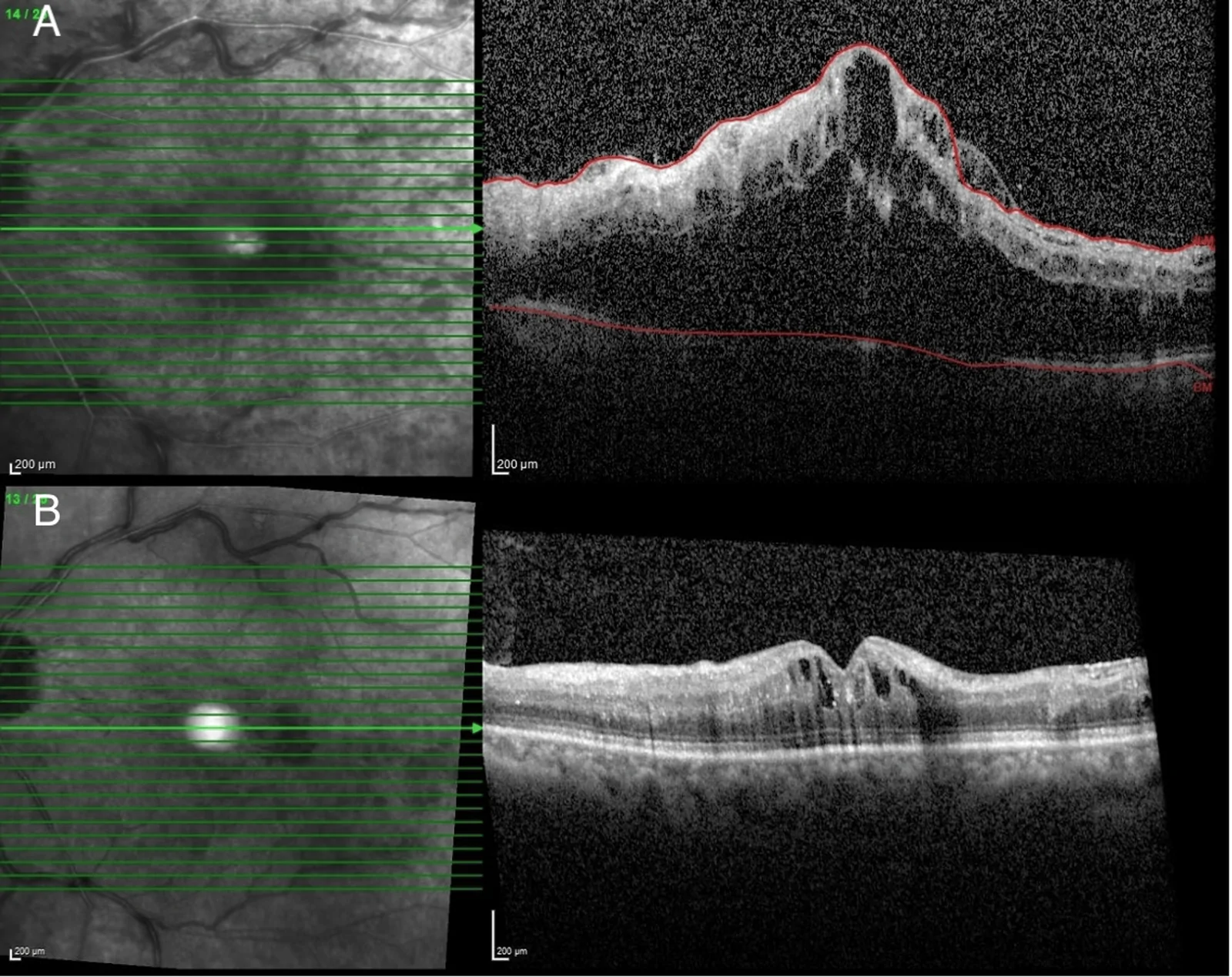
Optical coherence tomography of the left eye macula showing (A) cystoid macular oedema (CST 1100 µm) as a result of central retinal vein occlusion and (B) resolution with residual intraretinal fluid (CST 389 µm) after receiving loading doses of intravitreal anti-vascular endothelial growth factor injection

With the provisional diagnosis of nonischemic CRVO with CME, the patient received prompt intravitreal anti-VEGF therapy (ranibizumab: 0.5 mg), which was administered monthly for three consecutive months. At follow-up, her left eye BCVA improved to 6/24, anterior segment findings were unremarkable, and IOP remained in the low teens. Fundoscopy revealed a pink optic disc with resolving blot- and flame-shaped hemorrhages and no signs of ischemic conversion, such as neovascularization or vitreous hemorrhage (Figure 5). OCT showed marked resolution of CME, with CST reduced to 389 μm (Figure 6). Blood pressure control and lipid profile were optimized in collaboration with her physician, with emphasis on strict medication adherence. The chronological sequence of clinical events is summarized in Table 1.

**Table 1:** Table showing chronology of clinical events PXG: pseudoexfoliative glaucoma; IOP: intraocular pressure; ODR: ocular decompression retinopathy; CRVO: central retinal vein occlusion; BCVA: best-corrected visual acuity; CME: cystoid macula edema

Time	Clinical events
Initial presentation	Diagnosed with left PXG with IOP 62 mmHg, advanced glaucomatous optic neuropathy, and bilateral hypertensive retinopathy. Started on maximal medical therapy
Follow-up	IOP maintained at 15-20 mmHg on five topical antiglaucoma medications. Trabeculectomy was advised but declined
Lost to follow-up	Defaulted follow-up for 1 year and discontinued topical medications for 4 months
Re-presentation	Left IOP increased to 65 mmHg with glaucoma progression despite maximal medical therapy
26 April 2024	Trabeculectomy augmented with mitomycin C (0.02%) performed
Postoperative Day 1	Developed ocular hypotony (IOP: 5 mmHg) secondary to overfiltration due to conjunctival wound leak at suture site. Fundus findings were consistent with ODR
Postoperative Day 4	Conjunctival leak resolved following bandage contact lens application
6 weeks postoperatively	Diagnosed with nonischemic CRVO with CME
After diagnosis	Received three monthly intravitreal ranibizumab injections
Final follow-up	BCVA improved to 6/24, CME resolved, IOP remained stable, and no neovascular complications were observed

## Discussion

ODR has been reported following rapid IOP reduction in various types of glaucoma [1,3-6] and after multiple glaucoma procedures such as trabeculectomy [1,7], nonpenetrating glaucoma surgery [4], anterior chamber paracentesis [8], and laser iridotomy [6]. A study done in Seoul, South Korea, by Jung et al. reported an ODR incidence of 3.05%, and the most important risk factors for developing ODR are patients with high preoperative IOP and low hemoglobin [9]. Most reported cases are self-limiting with favorable visual outcomes following conservative management.

However, ODR followed by subsequent CRVO remains poorly described. Unlike the case reported by Huh and Jang, in which ODR only mimicked CRVO without angiographic evidence of venous occlusion [10], our patient subsequently developed true nonischemic CRVO confirmed by delayed AV transit on fluorescein angiography and significant CME. This case highlights the importance of distinguishing uncomplicated ODR from evolving CRVO during postoperative follow-up, as worsening retinal hemorrhages, increasing venous dilatation and tortuosity, delayed venous filling on fluorescein angiography, and persistent macular edema are not typical features of ODR alone and may require prompt anti-VEGF treatment. The coexistence of PXG, severe preoperative IOP elevation, poorly controlled hypertension, postoperative hypotony, and dyslipidemia may have collectively predisposed our patient to this rare sequential presentation.

Autoregulation of ocular blood flow stabilizes perfusion to the retina and optic nerve head despite fluctuations in ocular perfusion pressure (OPP) or metabolic demand, thereby preserving retinal homeostasis [11]. Experimental studies have further defined limits of autoregulatory failure. For instance, it was demonstrated that IOP as low as 6-7 mmHg marks the critical threshold below which macular blood flow can no longer be sustained, highlighting the retina’s vulnerability to hypotension [12]. Similarly, Puchner et al. found that retinal blood flow remains autoregulated until OPP falls by approximately 21 mmHg, beyond which perfusion declines sharply, emphasizing the delicate balance between OPP and vascular compensation [13]. In the present case, the inherent fragility of retinal vessels likely amplified these effects. Prolonged hypotony may reduce retinal arterial resistance, paradoxically increasing blood flow and promoting vascular leakage. This hemodynamic shift can overwhelm an already compromised endothelial barrier, intensifying fluid extravasation and contributing to the development of ODR.

Apart from the aforementioned factors, pseudoexfoliation syndrome may have predisposed the patient to the subsequent development of CRVO following ODR. PXG is typically asymmetrical and associated with higher and fluctuating IOP, reduced IOP tolerance, more advanced optic nerve damage at presentation, faster progression, and greater resistance to medical therapy often necessitating surgery [14]. Pathogenetically, pseudoexfoliation results from abnormal elastic microfibril aggregation, strongly linked to LOXL1 gene variants that disrupt elastic fiber homeostasis in the lamina cribrosa, increasing susceptibility to IOP-related injury [15]. OCT studies showed a thinner, posteriorly displaced lamina cribrosa in both nonglaucomatous pseudoexfoliation and PXG eyes compared to normal and primary open-angle glaucoma (POAG) eyes [16,17]. This structurally thinned lamina cribrosa may be more susceptible to further posterior displacement during prolonged hypotony, potentially impairing axoplasmic transport and resulting in optic disc edema. It has been hypothesized that the resultant optic nerve swelling could increase compression of the central retinal vein at the level of the lamina cribrosa, although this mechanism remains speculative.

The prognosis of ODR is generally favorable, with spontaneous resolution occurring within 2 to 72 weeks and complete visual recovery reported in approximately 85% of cases [2]. In the present case, however, visual recovery was incomplete due to progression to nonischemic CRVO complicated by CME. Although ODR often carries a good visual prognosis, proactive preventative measures are essential to avert its onset and reduce the risk of postoperative visual decline following glaucoma surgery. Preoperative precautions include performing surgery in a calm, controlled environment with well-managed IOP. Intraoperative strategies involve gradual IOP reduction, achieved by creating an oblique and controlled paracentesis; placing a small amount of viscoelastic in the AC prior to creation of sclerostomy; applying tight releasable sutures to prevent postoperative hypotony; and performing bleb titration at the conclusion of surgery, ensuring the AC remains formed with an adequately pressurized globe.

This case highlights the importance of careful postoperative retinal surveillance in patients with multiple vascular risk factors. Although ODR may mimic CRVO and is generally self-limiting, clinicians should remain alert for features suggestive of CRVO. Early retinal imaging and timely initiation of anti-VEGF therapy may improve visual outcomes when CRVO develops. As a single case report, this study cannot establish a causal relationship between ODR and subsequent CRVO. The development of CRVO was likely multifactorial, and extensive retinal hemorrhages may have obscured peripheral retinal ischemia on fluorescein angiography. Future studies are needed to determine the incidence of sequential ODR to CRVO after glaucoma surgery, identify patient- and procedure-related risk factors, and clarify the underlying hemodynamic and structural mechanisms linking these conditions. Prospective imaging studies evaluating changes in retinal perfusion and lamina cribrosa morphology following rapid IOP reduction may further improve understanding of this rare complication.

## Conclusions

This case describes a rare sequential occurrence of ODR followed by nonischemic CRVO after trabeculectomy in a patient with PXG and multiple vascular risk factors. While ODR is generally benign and self-limiting, clinicians should recognize that persistent or worsening retinal hemorrhages, venous dilatation, or macular edema may indicate sequential development to CRVO rather than uncomplicated ODR. Careful IOP control, prevention of postoperative hypotony, and vigilant retinal surveillance are essential to facilitate early diagnosis and timely treatment, thereby optimizing visual outcomes.
